# Matching the Directions of Electric Fields from Triboelectric and Ferroelectric Charges in Nanogenerator Devices for Boosted Performance

**DOI:** 10.1016/j.isci.2020.101011

**Published:** 2020-03-29

**Authors:** Andris Šutka, Kaspars Mālnieks, Linards Lapčinskis, Martin Timusk, Kaspars Pudzs, Martins Rutkis

**Affiliations:** 1 Research Laboratory of Functional Materials Technologies, Faculty of Materials Science and Applied Chemistry, Riga Technical University, Paula Valdena 3/7, 1048 Riga, Latvia; 2 Institute of Technical Physics, Faculty of Materials Science and Applied Chemistry, Riga Technical University, Paula Valdena 3/7, 1048 Riga, Latvia; 3 Institute of Physics, University of Tartu, W. Ostwaldi Str. 1, 50411 Tartu, Estonia; 4 Laboratory of Organic Materials, Institute of Solid State Physics, Kengaraga 8, 1063 Riga, Latvia

**Keywords:** Nanoparticles, Electromagnetic Field, Nanotechnology, Polymers, Devices

## Abstract

Embedding additional ferroelectric dipoles in contacting polymer layers is known to enhance the performance of triboelectricnanogenerator (TENG) devices. However, the influence of dipoles formed between the triboelectric surface charges on two contacting ferroelectric films has been ignored in all relevant studies. We demonstrate that proper attention to the alignment of the distinct dipoles present between two contacting surfaces and in composite polymer/BaTiO_3_ ferroelectric films can lead to up to four times higher energy and power density output compared with cases when dipole arrangement is mismatched. For example, TENG device based on PVAc/BaTiO_3_ shows energy density increase from 32.4 μJ m^−2^ to 132.9 μJ m^−2^ when comparing devices with matched and mismatched dipoles. The presented strategy and understanding of resulting stronger electrostatic induction in the contacting layers enable the development of TENG devices with greatly enhanced properties.

## Highlights

• TENG devices based on ferroelectric polymer/BaTiO_3_ composites were prepared
• $\vec{E}$ from ferroelectric dipoles and triboelectric charges drive electrostatic induction
• Matching $\vec{E}$ directions in TENG device lead to up to four times higher performance
• $\vec{E}$ from different sources are summing up and provide a stronger induction

## Graphical Abstract

**Figure:**
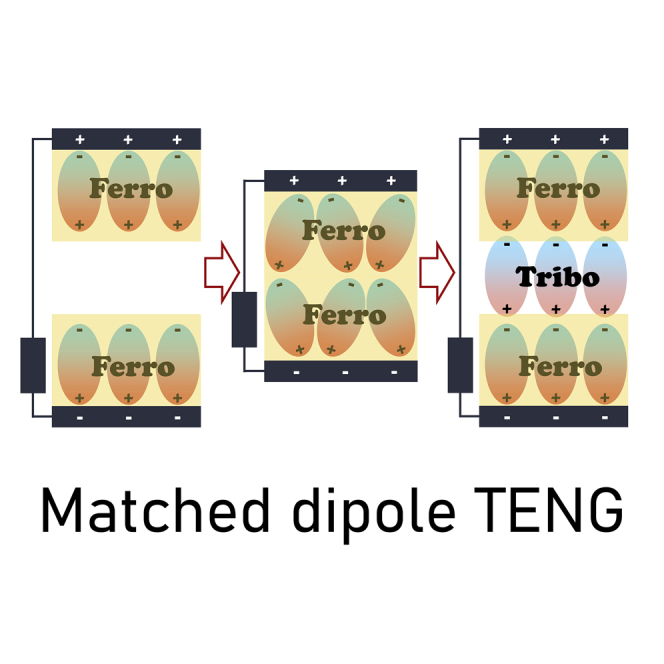

## Introduction

The field related to triboelectricnanogenerator (TENG) devices is emerging rapidly. Many original and creative TENG concepts have been presented in the literature for harvesting mechanical energy and converting it into electricity (Lee et al., 2019). The working principle of TENG devices is straightforward. The triboelectric materials (most commonly polymer insulators) are deposited on two conductive electrodes connected by an external circuit. Upon contacting-separating or sliding, surface charges are formed on the triboelectric materials, which induce an electrostatic charge on the conductive electrodes. Due to electrode oscillation or movement, a potential difference is created, which causes a current flow in the external electric circuit. TENG devices can be integrated into fabrics (Zhou et al., 2014), wearables (Kanik et al., 2015), interior objects (Dhakar et al., 2016), membranes (to harvest energy from sound) (Fan et al., 2015), and even implantable devices (Yao et al., 2018).

To enhance the performance of TENG, different approaches have been used. The most obvious way is to increase the specific contact area via nanostructuring (Zhang et al., 2015, Dudem et al., 2015, Zheng et al., 2014). Another possibility is the modification of surface or physicochemical properties of the triboelectric material (Šutka et al., 2019, Šutka et al., 2019, Lapčinskis et al., 2019, Fan et al., 2014, Yun et al., 2015, Wang et al., 2016). The performance can be also enhanced by using ferroelectric polymer or composite films as the contacting surfaces (Bai et al., 2014, Seung et al., 2017, Suo et al., 2016, Chun et al., 2015, Yang and Daoud, 2017, Choi et al., 2017, Lee et al., 2016, Šutka et al., 2018, Lapčinskis et al., 2019, Kim et al., 2019, Huang et al., 2020). State-of-the-art performance of ferroelectric material-based TENG devices can be expected when the ferroelectric material layers on contacting sides of the device are inversely polarized (Šutka et al., 2018, Lapčinskis et al., 2019). The contacted inversely polarized layers then act similarly to a series of connected capacitors (Lapčinskis et al., 2019). The total capacitance of the system decreases dramatically, whereas the potential difference increases as the air gap is created during separation. The induced charge redistribution in the external circuit manifests itself as current.

**Figure 1:**
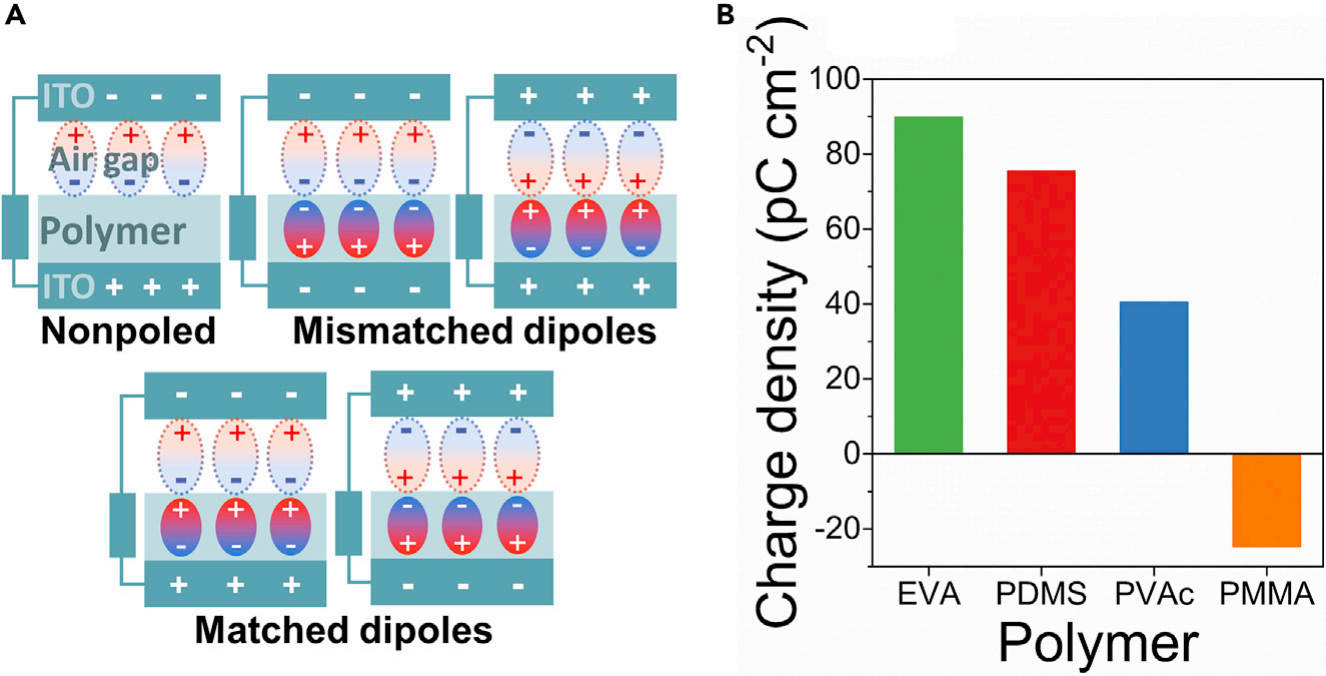
Dipole Formed between Polymer and ITO Can Be Matched with Ferroelectric Dipole Schematic representation of possible interactions between surface charge dipoles in the air gap and ferroelectric dipoles in TENG device (A). The calculated surface charge density of non-ferroelectric polymers indicates the magnitude and direction of the formed dipole in contact-separation cycle (B) (See also Figures S7 and S8).

However, previous works related to TENG devices based on ferroelectric materials overlook the dipole that forms between the triboelectric surface charges on contacting surfaces. As soon as we consider this additional factor, it follows that the electric field direction from ferroelectric dipoles should match the direction of the electric field from triboelectric surface charge to achieve maximum electric field strength and electrostatic induction. In other words, the alignment direction of ferroelectric dipoles has to coincide with the dipoles forming between the opposite charges on contacting surfaces as demonstrated in Figure 1A. In the present paper, we examine the performance of TENG devices when the electric field direction from surface charges and ferroelectric dipoles are matched or mismatched. To impart the ferroelectric properties for non-ferroelectric polymers used as triboelectric contacting layers, we added BaTiO_3_ nanoparticles. BaTiO_3_ were chosen as a feasible filler to allow preparation of ferroelectric polymer composite films so that contributions from contact-charging and ferroelectric dipoles could be compared. We demonstrate that matching the distinct kinds of dipoles present in TENG device leads to a significant increase in the power and energy density.

## Results and Discussion

Polydimethylsiloxane (PDMS), ethylene-vinyl acetate copolymer (EVA), poly(vinyl acetate) (PVAc), and poly(methyl methacrylate) (PMMA) were used in our studies to prepare TENG devices. The polymer films were spin coated on indium tin oxide (ITO) conductive electrode and contacted against another ITO (see Figure S1 for schematic TENG device representation). The polymer films were given ferroelectric properties by adding 7.5 vol% BaTiO_3_ nanoparticles <100 nm in size (see Figure S2 for scanning electron microscopy (SEM) image). Particles were well dispersed throughout the polymer matrix in the prepared composites (the SEM images of cross-sections for different composites are demonstrated inFigures S3–S6). The experimental details for sample preparation are provided in SI.

The sign of triboelectric surface charges formed on pure polymers after contacting against ITO was determined by measuring the current between the underlying electrode and the ground in Faraday cup mode Figure 1B (see also Figure S7). Polymers PDMS, PVAc, and EVA obtain a negative charge on their surface, whereas for PMMA a positive charge is observed when contacted ITO. The sign of the net triboelectric charge did not change when BaTiO_3_ nanoparticles were incorporated into the polymers (Figure S8).

All poled BaTiO_3_/polymer composites exhibit piezoelectric properties (Figure S9). The poling procedure is described in Supplemental Information. The piezoelectric charges of 2.9 pC cm^−2^, 10.9 pC cm^−2^, 3.7 pC cm^−2^, and 2.4 pC cm^−2^ were measured for BaTiO_3_/EVA, BaTiO_3_/PDMS, BaTiO_3_/PVAc, and BaTiO_3_/PMMA composites, respectively. The higher piezoelectric response of BaTiO_3_/PDMS could be attributed to its larger deformability under the constant loading force. The elastic modulus values of the four polymers are 5.48 GPa for PMMA, 5.24 GPa for PVAc, 44.3 MPa for EVA, and 2.9 MPa for PDMS (Šutka et al., 2019).

**Figure 2:**
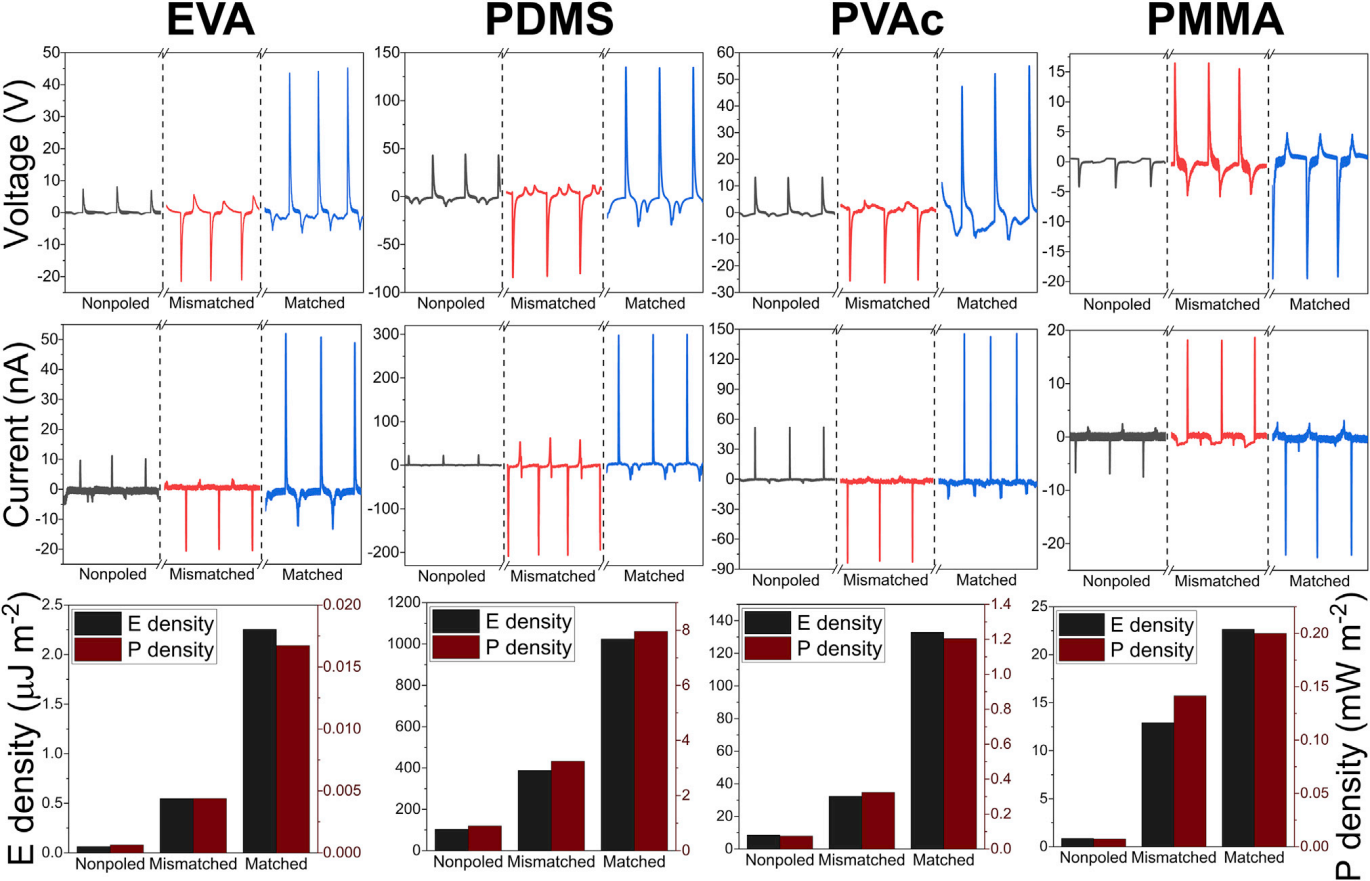
Comparison of Three Possible Dipole Alignment Configurations Ferroelectric dipoles are not present (non-poled), they are mismatched to dipoles formed by contact electrification (mismatched), and they are matched in the same direction as contact-electrification generated dipoles (matched). These states are shown to influence open-circuit voltage, short-circuit current, energy density, and power density for each BaTiO_3_/polymer composite studied in this work.

Figure 2 shows time-resolved current (I_SC_) and voltage measurements (V_OC_ at 1 GΩ) for TENG devices based on polymer composite layers and ITO. Composite layer in each of these TENG devices was tested as non-poled and also positively and negatively poled, so that ferroelectric dipole is matched and mismatched with the previously determined surface charge. The performance of a TENG device is very different depending on whether a positive or negative voltage is applied for poling (Figure 2), regardless of the polymer used. This phenomenon of one polarization direction resulting in a larger impact than the other is widely reported before (Bai et al., 2014, Suo et al., 2016, Lee et al., 2016, Šutka et al., 2018, Lapčinskis et al., 2019, Kim et al., 2019, Huang et al., 2020); however, the explanation provided for that has been inaccurate. We argue that the observed difference can be explained by elementary match and mismatch between the directions of the electric field from the ferroelectric dipole moment and the dipole moment created by triboelectric charges. As we can see from Figure 2, for PVAc, EVA, and PDMS, which obtain a negative triboelectric surface charge after contacting ITO, better performance is observed when the positive pole of the ferroelectric dipole is facing away from the underlying ITO electrode. In the case of PMMA, which has a positive triboelectric surface charge after contacting ITO, better performance is observed when the negative pole of the ferroelectric dipole is facing away from the substrate ITO electrode. Net dipole direction between surface triboelectric charges was confirmed by COMSOL finite element simulation (Figure S10). The experiments show that the TENG device has higher performance when the direction of dipole between triboelectric surface charge and ferroelectric dipoles are matching.

**Figure 3:**
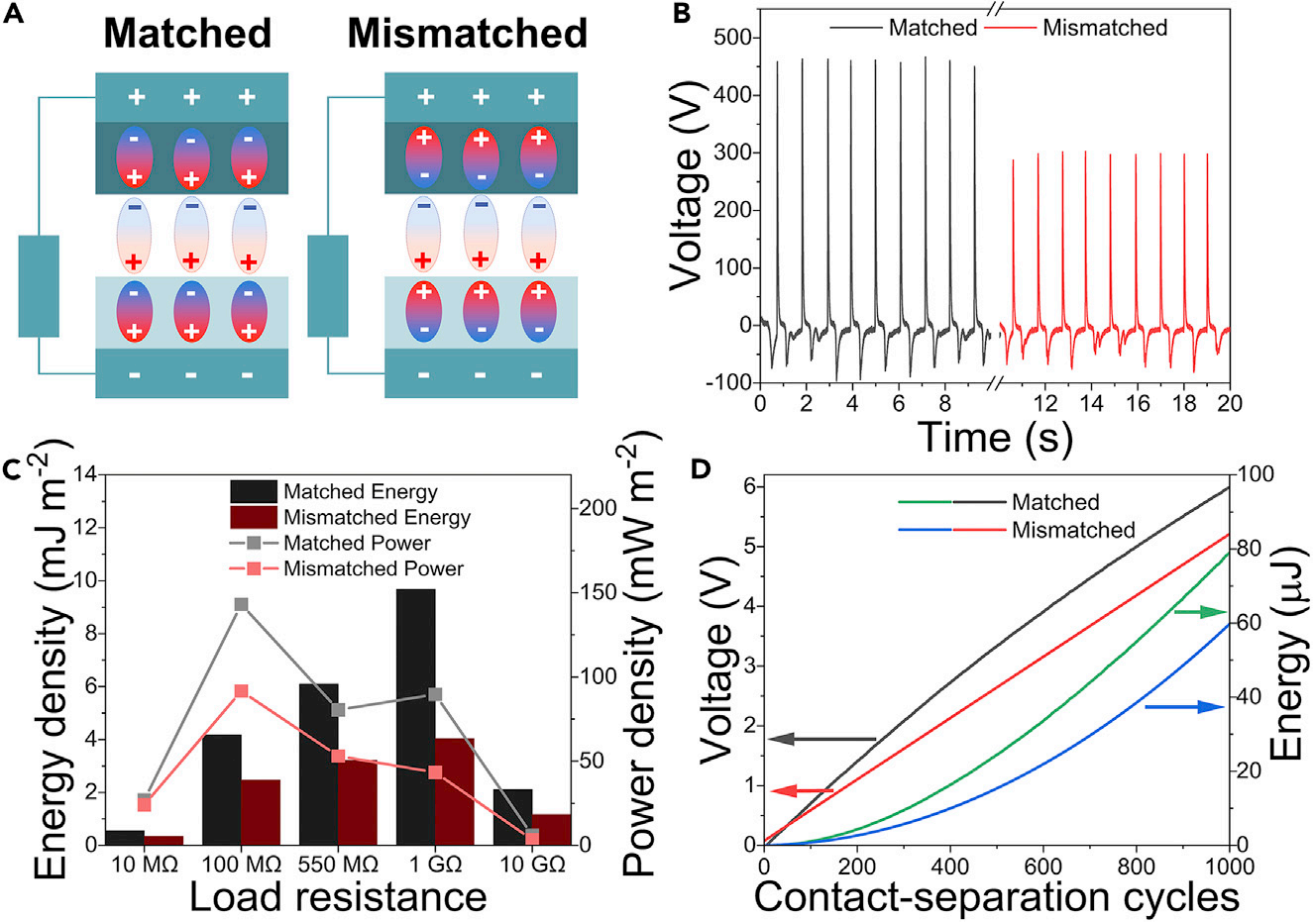
Comparison of TENG Devices Using Matched and Mismatched Dipole Configurations Inversely polarized BaTiO_3_/PDMS (top layer) and BaTiO_3_/PMMA (bottom layer) TENG device constructed in a way that ferroelectric polarization direction is matched (left) and mismatched (right) with contact-electrification-generated surface charge dipole direction (A). Graphs for matched and mismatched configuration show V_OC_ (at 1 GΩ) (B), energy, and power density during one contact-separation cycle (C) and the voltage across 4.4 μF capacitor during charging with the corresponding stored energy (D) (see also Figures S11–S13).

To provide even more evidence, we constructed a TENG device where both ITO electrodes are covered with a BaTiO_3_/polymer composite film. For the highest performance, the dipoles in the two films are polarized inversely (Šutka et al., 2018, Lapčinskis et al., 2019), whereas the polymer matrix material for each side is chosen so that the dipole moment direction of triboelectric and ferroelectric charges are matched. The surface charges on the contacted polymers used in the study were determined experimentally by measuring current in Faraday cup mode (Figure S11). A TENG device was constructed from PMMA and PDMS as PMMA charges positively and PDMS negatively. The poling direction in composite BaTiO_3_/PMMA was chosen so that the negative pole of the ferroelectric dipole was facing away from the substrate, whereas BaTiO_3_/PDMS was polarized so that the positive pole of ferroelectric dipole is facing away from the substrate. A schematic representation of the device is shown in Figure 3A. As expected, this particular TENG device performed excellently, and the output was superior to any other presented in this study. The peak open-circuit voltage (V_OC_) of device with matched dipoles reached 460 V as shown in voltage-time plot in Figure 3B. The instant energy and power densities of this TENG device reached 9.7 mJ m^−2^ and 143.2 mW m^−2^, respectively (black bars and gray line in Figure 3C). For comparison, a TENG device from the same polymers without BaTiO_3_ NPs shows V_OC_ as small as 16 V and three orders of magnitude smaller energy and power densities of 0.012 mJ m^−2^ and 0.104 mW m^−2^ (Figure S12). Also, the TENG device from the same inversely polarized composite films but with mismatched dipoles exhibited significantly lower output—V_OC_ of 300 V (red line in Figure 3B) and instant energy and power densities of 4.0 mJ m^−2^ and 91.6 mW m^−2^ from the same contacting area (red bars and red line in Figure 3C).

**Figure 4:**
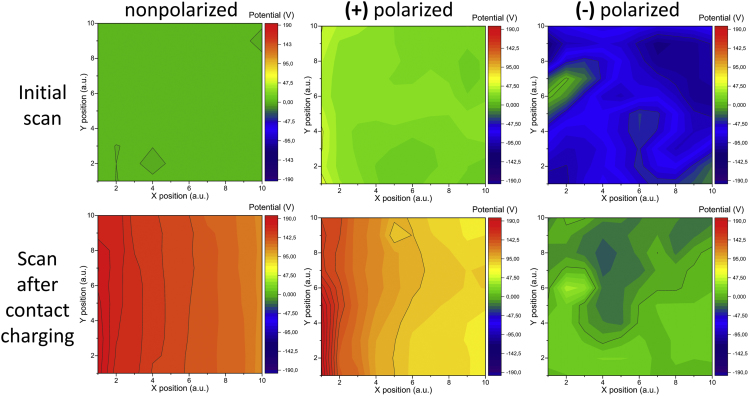
Surface Potential Maps of Three BaTiO_3_/PDMS Composites (Nonpolarized, Positively and Negatively Polarized) Top maps show respectively neutral, positive, and negative surface potential at initial scan. All surfaces show tendency to charge positively after contact with PMMA as evidenced by more positive surface potential (bottom). A gradual decrease of potential in the direction from left to right is observed because the triboelectric charge decreases as the surface is being scanned in this direction.

We also performed a macroscale scanning Kelvin Probe measurements (detailed description given in Transparent Methodssection). The average surface potential for nonpolarized BaTiO_3_/PDMS was determined to be 0.06 V. The average potential after contacting with PMMA was measured to be 146.4 V (surface potential maps of a nonpolarized sample before and after contacting PMMA are shown in Figure 4). Surface potential scanning was also performed on both positively and negatively polarized BaTiO_3_/PDMS samples before and after contacting with PMMA. Average potential values at first were measured to be 24.3 V and −70.5 V, respectively. A quasi-permanent surface potential is created by ferroelectric dipoles in PDMS/BaTiO_3_ composite layer. Just like in the case of a nonpolarized sample, contacting PMMA caused both samples to obtain an additional positive charge. Therefore, the average potential value grew to 107.2 V for the positively polarized sample and to −1.1 V in the case of the negatively polarized sample after they were contact separated. Full-surface potential maps for both polarized samples before and after the contact with PMMA are shown in Figure 4. Experimental results confirm the proposed mechanism for better performance of TENG devices where two sources of the electric field are present—triboelectric charges and ferroelectric dipoles. In the case of the matched direction of electric fields arising from triboelectric and ferroelectric dipoles, the electric field strength summarizes and amplifies the electrostatic induction on TENG electrodes. Due to the potential difference between positive and negative induced charge, the stronger current flow between two TENG electrodes is measured. As shown in Figure S11B, a PDMS contact layer induces a positive current peak on the underlying electrode when contacted against PMMA. Therefore, surface potential of positively polarized PDMS layer (positive pole of ferroelectric dipole facing away from underlying electrode) after contact with PMMA corresponds to a matched dipole case, whereas the negatively polarized corresponds to mismatched dipoles.

The TENG device that was constructed from inversely polarized BaTiO_3_/PDMS and BaTiO_3_/PMMA composite films with matched dipoles was used to charge a 4.4 μF capacitor (Figure S13). This experiment is beneficial to understanding the practicality of the developed device because charged capacitors can be further used to charge batteries or power small sensors, and voltage across the capacitor also conveniently shows the amount of stored energy. By performing 1,000 contact-separation cycles the voltage across the capacitor increased by 6.0 V that corresponds to the stored energy of 79 μJ, as demonstrated in Figure 3D. In the meantime, TENG device constructed from equivalent contact layers, but with intentionally mismatched dipoles, increased the voltage across the capacitor only by 5.2 V and accumulated 60 μJ energy under the same operating conditions and number of contact-separating cycles.

The proof-of-concept demonstrated here can be further used to construct TENG devices with record performance. Polymers with a superior tendency for triboelectrification (Lapčinskis et al., 2019) and polymer composite materials with high intrinsic piezoelectric responses have been reported in literature widely (Zhang et al., 2018, Jeong et al., 2019, Han et al., 2019). A combination of methodology shown in this paper with high-performance ferroelectric materials could bring the TENG research community closer to new cutting-edge discoveries.

### Conclusions

Results presented here shine a light upon the path to improvements in the construction of advanced TENG devices. The energy and power density of a TENG device can be increased up to four times by matching the directions of electric fields arising from ferroelectric dipoles and triboelectric surface charges when compared with mismatched case. Matching of the dipoles magnifies the electric field strength and electrostatic induction on a conductive electrode that in turn leads to a higher voltage output.

### Limitations of the Study

Performance values (V_OC_, I_SC_, Energy, and power density) in this work do not reach state-of-the-art level reported in the latest works. However, this study shows proof of concept that would allow to supersede all reported TENG devices. This could be done by more elaborate choice of polymer materials with superior contact-charging capabilities and by choosing ferroelectric materials with much higher piezoelectric constant d_33_.

## Methods

All methods can be found in the accompanying Transparent Methods supplemental file.

## Supplemental Information

Document S1. Transparent Methods and Figures S1–S13

## Footnotes

Supplemental Information can be found online at https://doi.org/10.1016/j.isci.2020.101011.
