# Supplementary material for: Matching the Directions of Electric Fields from Triboelectric and Ferroelectric Charges in Nanogenerator Devices for Boosted Performance
Source: iScience. 2020 Mar 29;23(4):101011. doi: 10.1016/j.isci.2020.101011 (PMC7138923; doi:10.1016/j.isci.2020.101011)
Supplement: Document S1. Transparent Methods and Figures S1–S13 [file mmc1.pdf]

iScience, Volume *23*

Supplemental Information

Matching the Directions of Electric Fields from Triboelectric and Ferroelectric Charges in Nanogenerator Devices for Boosted Performance

Andris Šutka, Kaspars Mālnieks, Linards Lapčinskis, Martin Timusk, Kaspars Pudzs, and Martins Rutkis

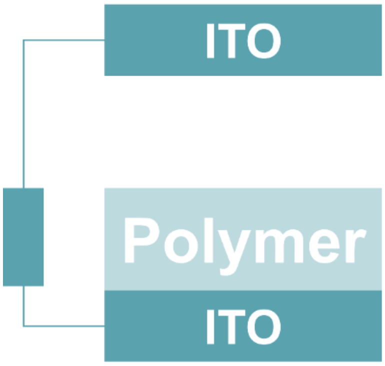

Figure S1, related to Figure 1 (a). Simplified schematic TENG device.

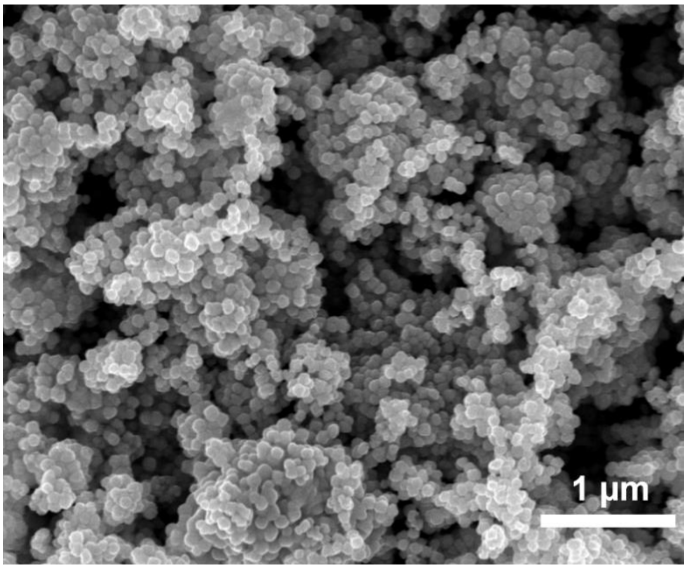

Figure S2, related to Figure 1 (a). SEM image of commercial $BaTiO_3$ nanoparticles (Sigma-Aldrich, CAS number 467634) used for sample film properties.

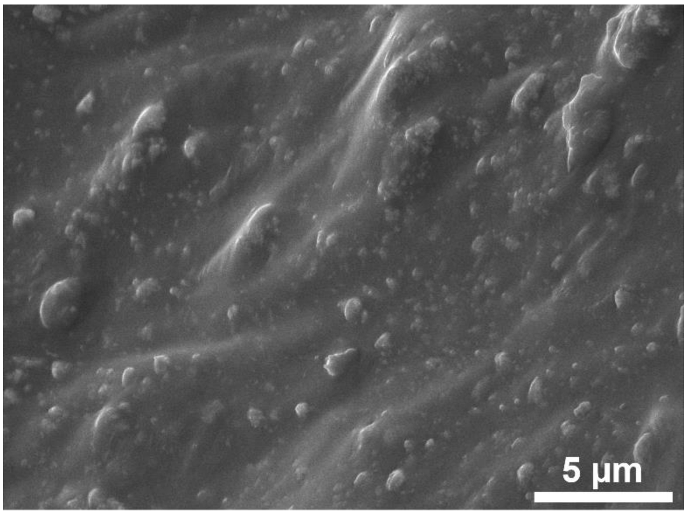

Figure S3, related to Figure 1 (a). SEM image of $BaTiO_3$/PDMS composite film cross-section.

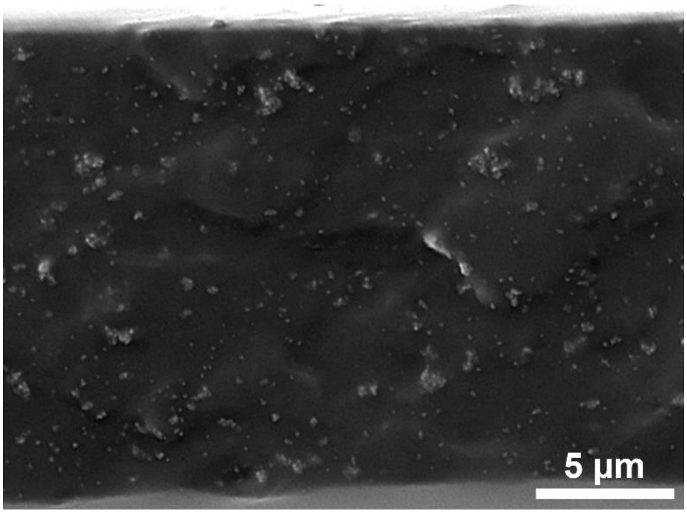

Figure S4, related to Figure 1 (a). SEM image of $BaTiO_3$/EVA composite film cross-section.

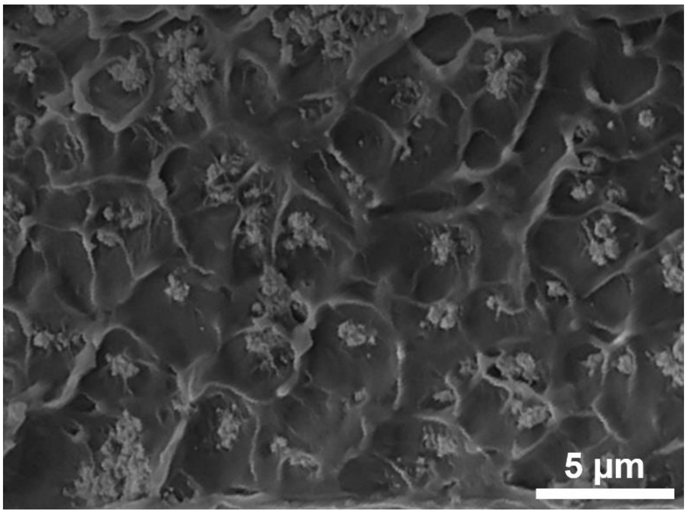

Figure S5, related to Figure 1 (a). SEM image of $BaTiO_3$/PVAc composite film cross-section.

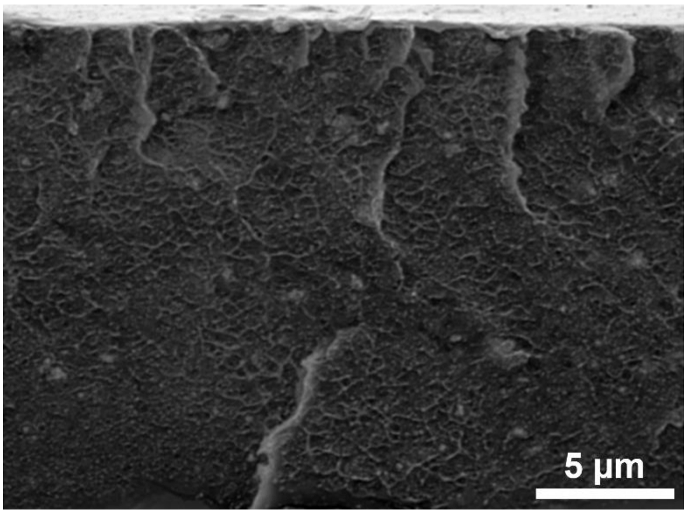

Figure S6, related to Figure 1 (a). SEM image of $BaTiO_3$/PMMA composite film cross-section.

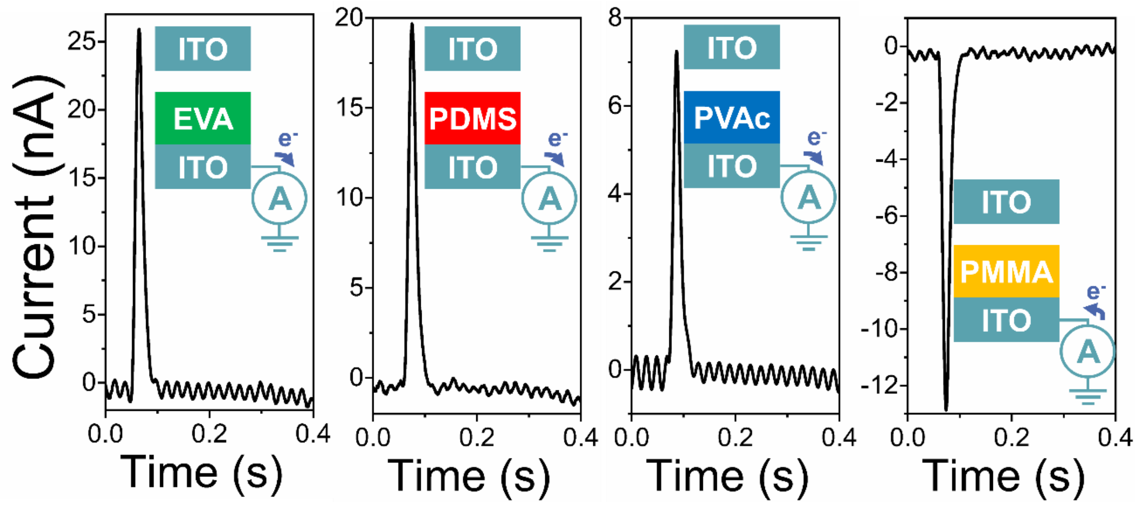

Figure S7, related to Figure 1 (b). Faraday cup regime measurements of EVA, PDMS, PVAc and PMMA against ITO with the corresponding schemes.

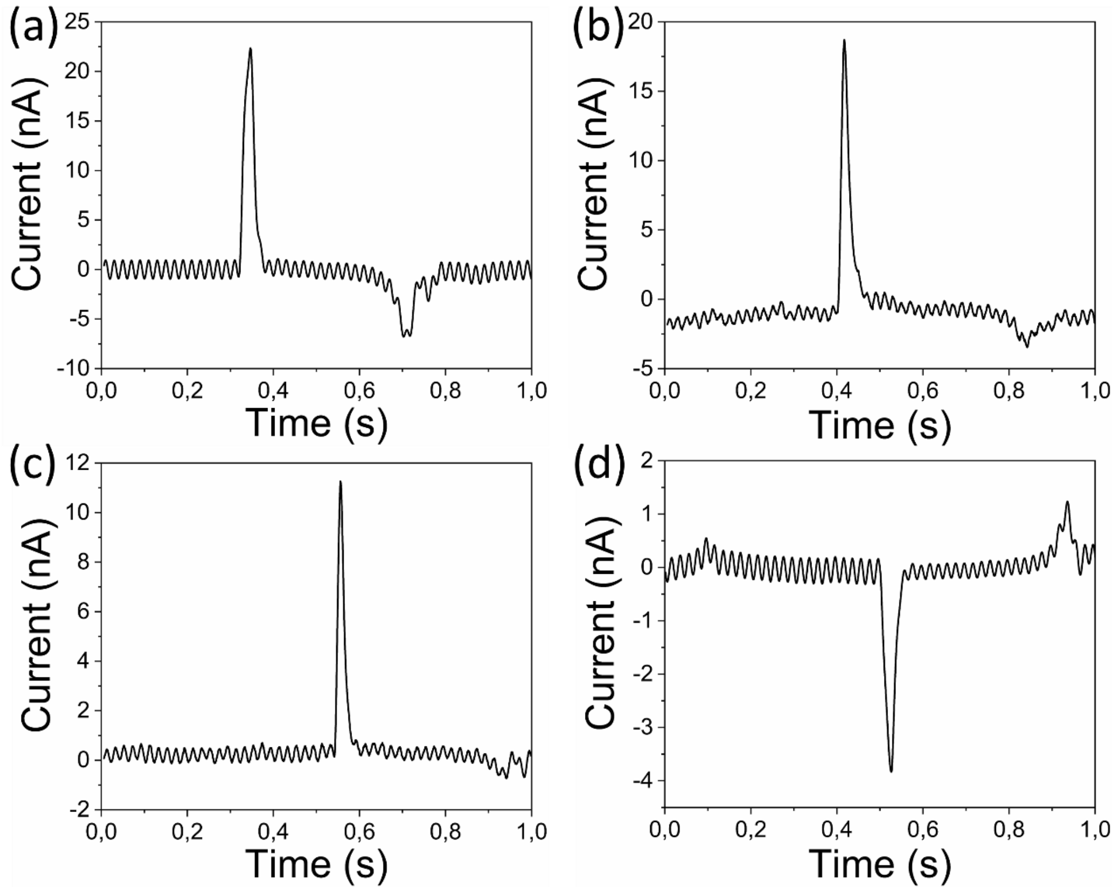

Figure S8, related to Figure 1 (b). Faraday cup regime measurements of (a) $BaTiO_3$/EVA, (b) $BaTiO_3$/PDMS, (c) $BaTiO_3$/PVAc and (d) $BaTiO_3$/PMMA composites against ITO.

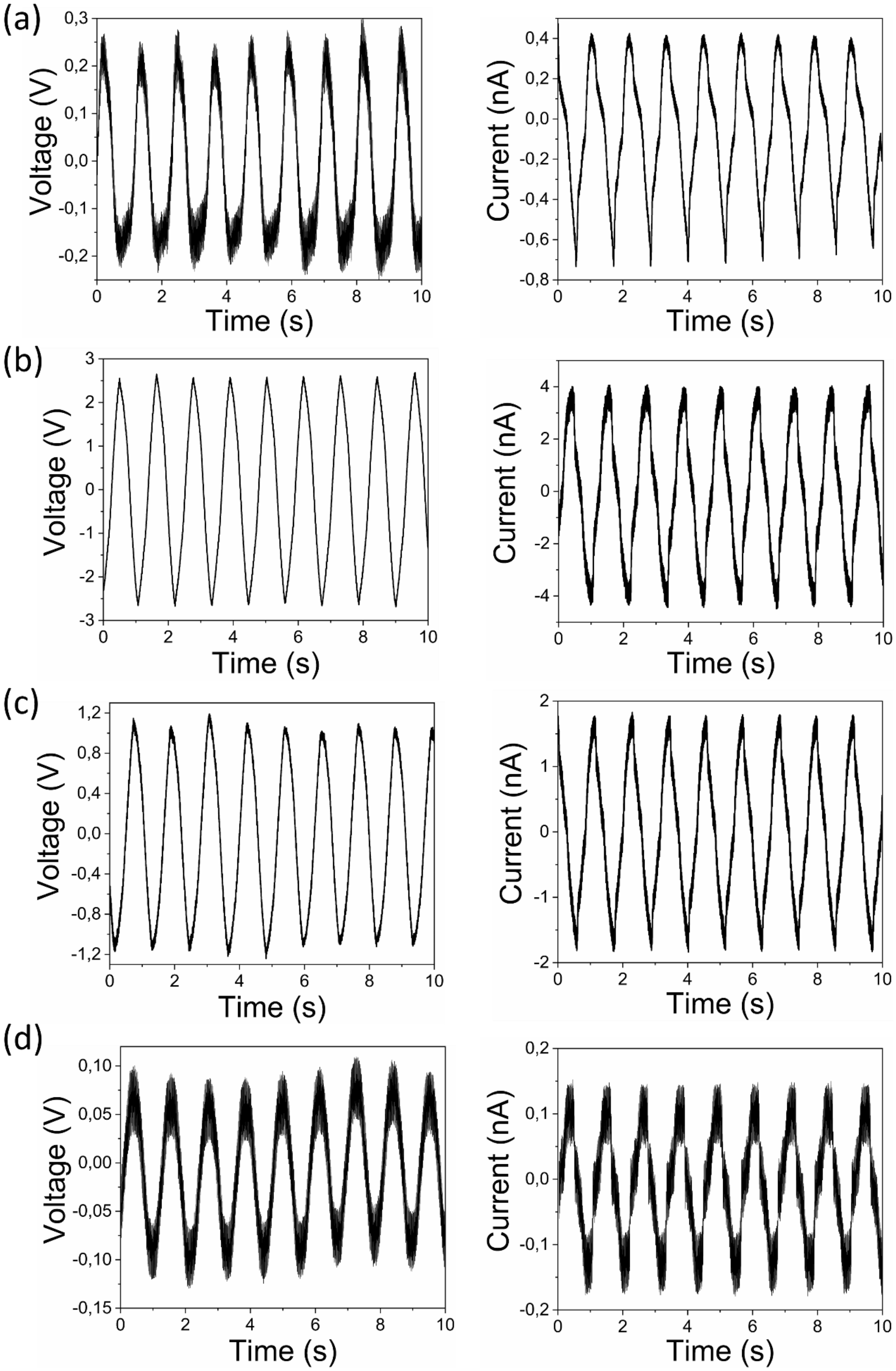

Figure S9, related to Figure 1 (a). Piezoelectric V_{OC} and I_{SC} of (a) $BaTiO_3$/EVA, (b) $BaTiO_3$/PDMS, (c) $BaTiO_3$/PVAc and (d) $BaTiO_3$/PMMA composites.

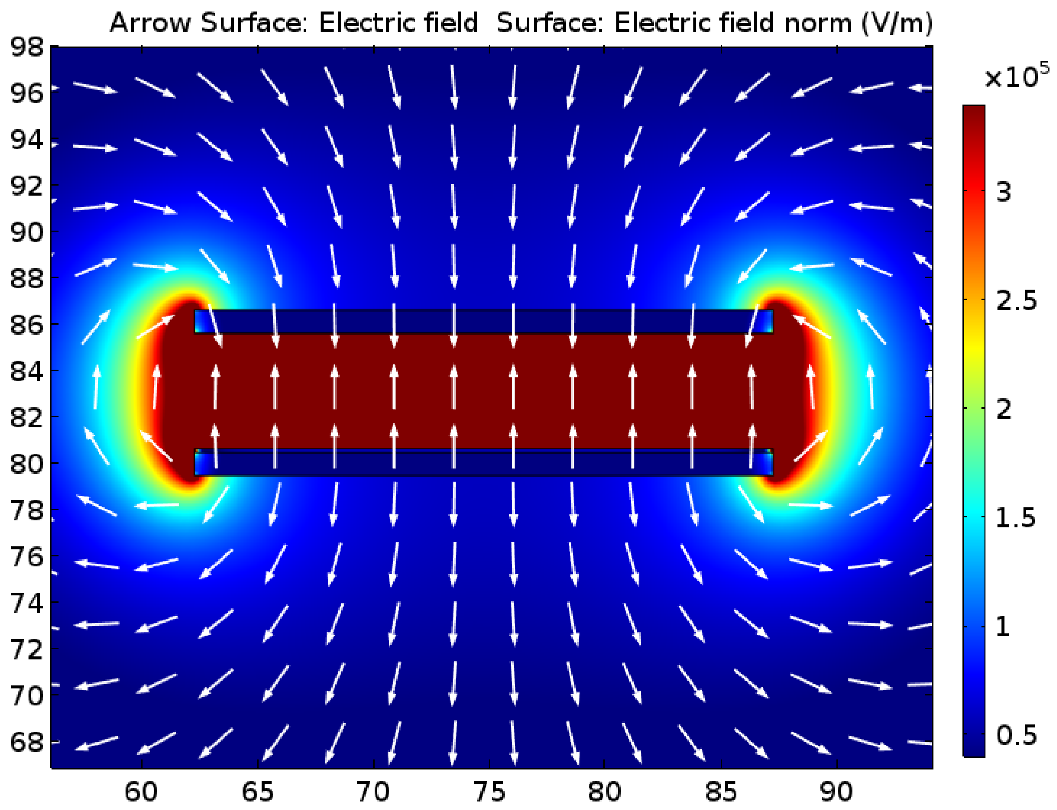

Figure S10, related to Figure 2. COMSOL finite element simulation of contact-separation between two films. The dipole formed between PDMS and opposite layer is shown in the middle where the highest electric field is observed (3.4×10^5 V m^{-1}).

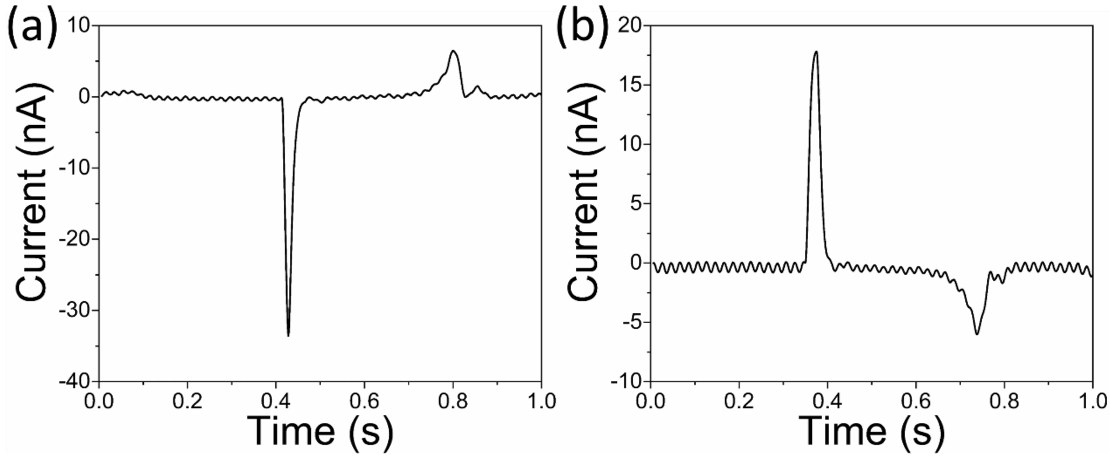

Figure S11, related to Figure 3 (a). Faraday cup regime measurements of (a) PMMA sample contacted with PDMS layer and (b) PDMS sample contacted with PMMA layer.

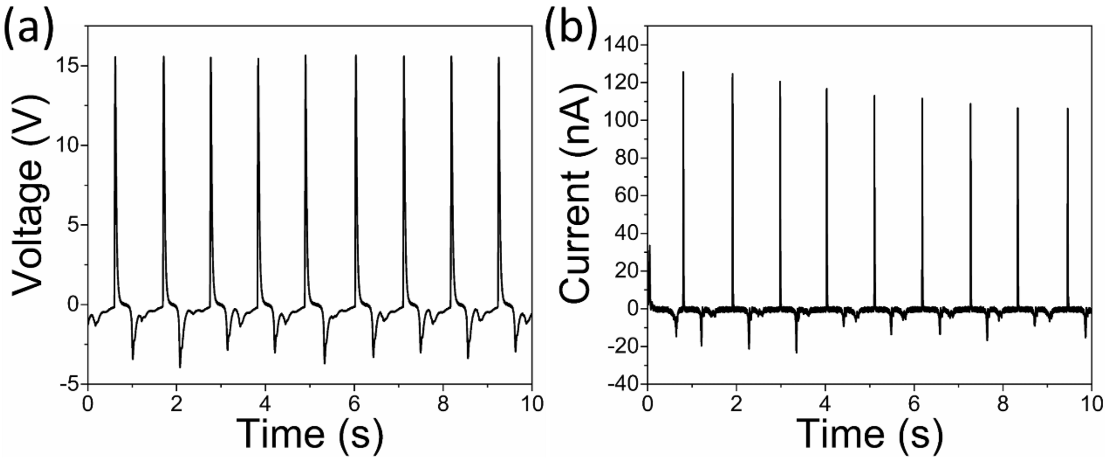

Figure S12, related to Figure 3 (b). (a) V_{OC} and (b) I_{SC} of TENG device constructed from PDMS and PMMA polymers without $BaTiO_3$ NPs.

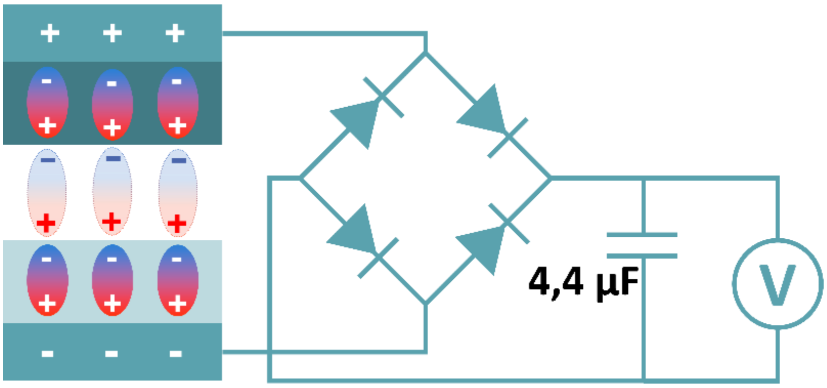

Figure S13, related to Figure 3 (d). Capacitor charging scheme. Alternating current signal from TENG device is rectified and energy is stored in 4.4 µF capacitor. Voltage is measured to determine the charge state of capacitor.

Transparent methods

Polydimethylsiloxane (PDMS), ethylene-vinyl acetate copolymer (EVA), poly(vinyl acetate) (PVAc) and poly(methyl methacrylate) (PMMA) were used in our studies to prepare TENG devices. The polymer film was deposited on indium-tin oxide (ITO) coated polyethylene terephthalate (PET) substrate by spin-coating and contacted with another ITO (see Figure S1 for schematic TENG device representation). Polydimethylsiloxane (PDMS, Sylgard 184 kit) films were prepared by mixing base polymer and curing agent in a 10:1 ratio. PDMS films were spin-coated directly on ITO conductive electrode. The rotation speed was 3000 rpm. Before spin coating, mixture was degassed and PDMS films were cured at 80°C for 30 min. Polymers EVA (Sigma Aldrich, 40 wt% vinyl acetate) and PVAc (Sigma Aldrich, average Mw 100`000) were dissolved in toluene in 20 wt%, while dimethylformamide was used for PMMA (Sigma Aldrich, average Mw 120`000). Solutions were spin-coated onto ITO/PET at 3000 rpm and dried in ambient atmosphere to obtain polymer films. The ferroelectric properties for polymer films were provided by adding 7.5 vol% $BaTiO_3$ nanoparticles <100 nm in size (see Fig. S2 for scanning electron microscopy (SEM) image). Nanoparticles were dispersed in polymer solutions using ultra sonification probe Hielscher UP200S for 3 minutes. The cross-section images for composites are demonstrated in Fig. S3-S6. Polarization of prepared $BaTiO_3$ and polymer composite films was conducted using 20 MV m^{-1} electric field at 90 °C temperature.

The open-circuit voltage (V_{OC}) at load resistance $1{\cdot}10^9$ Ω and short-circuit current (I_{SC}) was measured by using a custom-made voltage divider in combination with a Keithley 6514 electrometer connected to a Picoscope 5444B PC oscilloscope to provide high time resolution. The surface charges were calculated from current measured in Faraday cup regime between TENG electrode and ground (shown in Figure S7) by using equation $Q=\int idt$ (where i – instantaneous current). Contacting area (sample size) in all tests was 5 cm^2. Pressing force (10 N), frequency (1 Hz), separation speed (10 mm s^{-1}) and gap (5 mm) between sample sides for TENG performance measurements were kept constant by using INSTRON E1000 All-Electric Dynamic Test Instrument. The instantaneous power density was calculated by Joule's Law: $P=V^2R^{-1}$, where V is voltage and R is load resistance. Integration of instantaneous power density over time gives energy density obtained during contact-separation. Next, energy density divided by the duration of the peak results in the average power density. The 4.4 μF capacitor was charged as depicted in Figure S13. Energy stored in this capacitor was calculated by $E=0.5CV^2$ (C is capacitance of capacitor and V is voltage measured across capacitor).

Kelvin probe measurements were performed using Scanning Kelvin Probe system SKP5050. Surface potential of the sample was scanned by oscillating 2 mm tip in 10-by-10 point area (total 100 points) to obtain surface potential maps. Distance between two scanned points was approximately 250 μm, therefore almost the whole area of sample was scanned. Potential maps of non-polarized and polarized (both positively and negatively) $BaTiO_3$/PDMS samples before and after contacting with PMMA polymer is shown in Figure 4 in main text. The contact with PMMA was accomplished by placing this layer on $BaTiO_3$/PDMS sample still in device and applying load of 10 N for 1 minute. Second scan was performed right after the removal of PMMA.

The electric field strength and gradient were simulated for contacting-separating PDMS film with ITO by using COMSOL finite element analysis software. Charge density used for electric field simulations was calculated from measured current in Faraday cup mode by equation $Q=\int idt$ (where i – instantaneous current). Simulation results are presented in Figure S10.
